# The Cancer-Testis Long Non-coding RNA PCAT6 Facilitates the Malignant Phenotype of Ovarian Cancer by Sponging miR-143-3p

**DOI:** 10.3389/fcell.2021.593677

**Published:** 2021-02-04

**Authors:** Xiaofang Tan, Yang Shao, Yue Teng, Siyu Liu, Weijian Li, Lu Xue, Yuepeng Cao, Chongqi Sun, Jinhong Zhang, Jing Han, Xiaoli Wu, Hanzi Xu, Kaipeng Xie

**Affiliations:** ^1^Nanjing Maternity and Child Health Care Hospital, Women's Hospital of Nanjing Medical University, Nanjing, China; ^2^Jiangsu Cancer Hospital, Jiangsu Institute of Cancer Research, The Affiliated Cancer Hospital of Nanjing Medical University, Nanjing, China; ^3^The First Affiliated Hospital of Nanjing Medical University, Nanjing, China; ^4^Maternal and Child Care Service Center, Nanjing, China

**Keywords:** PCAT6, ovarian cancer, miR-143-3p, TAK1, ceRNA

## Abstract

**Background:** It has been reported that long non-coding RNAs (lncRNAs) play critical roles in tumorigenesis. However, their roles in ovarian cancer (OC) remain to be elucidated. The aim of this study was to uncover the function and underlying mechanisms of PCAT6 in OC.

**Methods:** The expression pattern of PCAT6 in OC was analyzed in the GSE137238, GSE143897 and Gene Expression Profile Interactive Analysis (GEPIA) datasets. Kaplan–Meier Plotter online software was used for survival analysis. Loss-of-function assays and gain-of-function assays were used to assess the function of PCAT6 in OC development. Moreover, small-RNA sequencing, bioinformatic analysis, luciferase assays and rescue experiments were carried out to clarify the potential mechanism of PCAT6 in OC.

**Results:** PCAT6 expression was significantly increased in OC tissues and positively correlated with advanced stages and with poor overall survival, progression-free survival and post-progression survival. Knockdown of PCAT6 in A2780 and SKOV3 cells inhibited OC cell proliferation, migration and invasion. In contrast, Overexpression of PCAT6 exerted the opposite effects on OC cells. Notably, PCAT6 bound to miR-143-3p and affected the expression of transforming growth factor (TGF)-β-activated kinase 1 (TAK1). Subsequent rescue assays confirmed that upregulation of miR-143-3p decreased the PCAT6 overexpression-induced promotion of proliferation, migration and invasion. Moreover, downregulation of miR-143-3p reversed the PCAT6 knockdown-induced inhibition of proliferation, migration, and invasion.

**Conclusions:** Our findings demonstrate that PCAT6 plays an oncogenic role in OC and may be useful as a therapeutic target for OC.

## Introduction

Ovarian cancer ranks as the seventh most common malignancy and the second most common cause of gynaecologic cancer death in women, with 239,000 new cases of ovarian cancer and 152,000 deaths annually worldwide (Bray et al., 2018). The early symptoms of ovarian cancer are not specific or pathognomonic, and the majority of cases are diagnosed at an advanced stage with widespread peritoneal dissemination throughout the abdomen and pelvis (Lheureux et al., 2019). Although cytoreductive surgery and platinum-taxane chemotherapy have increased the survival times of ovarian cancer patients, the 5-year survival rate for patients with metastatic epithelial ovarian cancer is <30% (Reavis and Drapkin, 2020). Given the high metastasis and recurrence rates of ovarian cancer, considerable effort should be made to find novel effective targets for early diagnosis and therapy of this disease.

In recent years, the number of scientific studies related to cancer-associated long non-coding RNAs (lncRNAs) has exponentially increased (Tripathi et al., 2018). Similar to coding genes, various lncRNAs can be classified as tumor suppressor genes and oncogenes according to their expression patterns and functions in cancer biology (Shi X. et al., 2018; Yeh et al., 2018; Hu et al., 2019; Yang et al., 2019). Previous studies have demonstrated that cancer-associated lncRNAs can act as competitive endogenous RNAs (ceRNAs) by binding to miRNAs, thus modulating the derepression of miRNA targets (Liang et al., 2018; Dong et al., 2019; Gokulnath et al., 2019; Wu et al., 2019). For example, MAGI2-AS3 has been reported to play a tumor-suppressive role in high-grade serous carcinoma (HGSC) by sponging miR-15-5p, miR-374a-5p, and miR-374b-5p (Gokulnath et al., 2019). Dong et al. found that HOXD-AS1 promotes ovarian cancer cell migration and invasion through the HOXD-AS1/miR-186-5p/PIK3R3 pathway (Dong et al., 2019). Findings by Liang et al. demonstrate that overexpression of PTAR can facilitate metastasis by regulating miR-101 in ovarian cancer cells, whereas downregulation of PTAR attenuates TGF-β1-induced carcinogenesis (Liang et al., 2018). Overall, the evidence indicates that it is essential to elucidate the molecular mechanisms of dysregulated lncRNAs in ovarian cancer. Knowledge of these mechanisms will contribute to understanding of ovarian cancer pathogenesis and exploration of therapeutic targets for the disease.

The intergenic lncRNA prostate cancer-associated transcript 6 (PCAT6), located at 1q32.1, has been reported to play oncogenic roles in multiple cancers. Wan et al. demonstrated that the expression level of PCAT6 is significantly increased in lung cancer tissues and cells and that PCAT6 knockdown inhibits cellular proliferation and metastasis (Wan et al., 2016). Moreover, PCAT6 can facilitate the proliferation and metastasis of cervical cancer cells but suppress apoptosis via the PCAT6/miR-543/ZEB1 axis (Ma et al., 2020). Additionally, Dan et al. determined that the PCAT6 expression levels in extracellular vesicles are significantly increased in the peripheral blood of lung cancer patients (Bai et al., 2019). One recent study has shown that PCAT6 might promote the malignancy of ovarian cancer cells by inhibiting PTEN (Kong et al., 2019). However, the detailed regulatory mechanism of PCAT6 remains to be fully elucidated. Furthermore, other underlying mechanisms of PCAT6 in ovarian cancer development should be explored.

In our research, we found that PCAT6 expression is upregulated in ovarian cancer tissues and is associated with clinical stage and patient survival. Loss-of-function and gain-of-function assays showed that PCAT6 induces cell proliferation, invasion and migration in ovarian cancer cells. We further revealed that PCAT6 acts as a ceRNA to regulate transforming growth factor (TGF)-β-activated kinase 1 (TAK1) expression by binding to miR-143-3p. Together, our findings indicate that PCAT6 is a potential ovarian cancer diagnostic and prognostic biomarker and that the PCAT6-miR-143-3p-TAK1 axis participates in proliferation, migration and invasion in ovarian cancer. Our results may provide novel insight that will aid in the development of therapeutic targets for ovarian cancer.

## Materials and Methods

### PCAT6 Expression in Ovarian Cancer From Public Online Datasets and Survival Analysis

PCAT6 expression and clinical characteristics in ovarian cancer from the GSE137238 and GSE143897 datasets were downloaded from the Gene Expression Omnibus (GEO, https://www.ncbi.nlm.nih.gov/geo/) database. GSE137238 consisted of data for eight pairs of primary ovarian tumors and matched normal fallopian tubes. GSE143897 included data for 11 benign tissues, 79 serous ovarian cancer tumors and 32 ascites. PCAT6 expression was log2-transformed for further analysis. The expression levels of PCAT6 in ovarian cancer and normal ovarian tissues were obtained with the Gene Expression Profile Interactive Analysis (GEPIA) web server (http://gepia.cancer-pku.cn/index.html). Moreover, the associations between PCAT6 and overall survival (OS), progression-free survival (PFS) and post-progression survival (PPS) in ovarian cancer patients were analyzed with Kaplan–Meier Plotter (http://kmplot.com/analysis/index.php?p=service&cancer=ovar). The patients were grouped by the median PCAT6 expression, and the hazard ratios (HRs) with 95% confidence intervals (CIs) were calculated.

### Cell Culture

The human ovarian cancer cell lines SKOV3 and A2780 were purchased from Shanghai Zhong Qiao Xin Zhou Biotechnology Co., Ltd. The HEK293 human embryonic kidney cell line (HEK293T) was a kind gift from Professor Chenbo Ji (Nanjing Medical University, Nanjing, China). The SKOV3 cells were cultured in McCoy's 5a medium (Gibco, Grand Island, NY, USA) supplemented with 10% fetal bovine serum (FBS). The A2780 and HEK293T cells were cultured in Dulbecco's modified Eagle's medium (DMEM, KeyGEN BioTECH, Nanjing, China) supplemented with 10% FBS. All cell lines were maintained in a humidified atmosphere containing 5% CO_2_ at 37°C.

### Transfection

LncRNA PCAT6 Smart Silencer, miR-143-3p mimics and a negative control (NC) sequence were designed and synthesized by RiboBio, Inc. (Guangzhou, China). PCAT6 plasmids were synthesized by Genecreate Biological Engineering Co., Ltd. (Wuhan, China) and verified by sequencing. Cells were seeded into six-well plates (Costar, USA), and Lipofectamine 2000 (Invitrogen, Grand Island, NY, USA) was utilized to conduct transfection according to the manufacturer's protocols. After 48 h of culture, cells were harvested for RNA extraction.

### Cell Proliferation

Cells (1 × 10^3^/well) were seeded into 96-well plates 24 h after transfection. Proliferation was tested using a Cell Counting Kit-8 (CCK-8) (Dojindo Molecular Technologies, Inc., Shanghai, China) in accordance with the manufacturer's protocol. CCK-8 reagent was added immediately after cell adhesion (marked as 0 h) and at 12, 24, 36, 48, and 72 h after cell adhesion. The absorbance was measured 1.5 h after CCK-8 was added, and the cell proliferation curves were plotted according to the results above.

### Cell Migration and Invasion Assay

Cell invasion and migration were analyzed using Transwell chambers (Costar) coated or not coated with Matrigel (BD Biosciences, CA). After this step, 5 × 10^4^ cells in 200 μl of serum-free medium were seeded into the upper chamber (for the cell invasion assay, the chamber was coated with Matrigel diluted 1:7 in McCoy's 5a Medium for SKOV3 and DMEM for A2780 cells). To the lower chamber, 600 ul of McCoy's 5a medium for SKOV3 cells or 600 ul of DMEM for A2780 cells supplemented with 20% FBS, 100 ug/ml streptomycin and 100 U/ml penicillin was added. The cells were incubated for 48 h for migration and 72 h for invasion at 37°C with 5% CO_2_, after which the cells on the upper surface were removed. The cells on the lower surface were fixed with 4% paraformaldehyde for 30 min and stained with 0.1% crystal violet for 15 min. After removing the cells from the upper surface of the chamber, images were obtained under a microscope.

### Wound Healing Assays

Cells were seeded into 6-well plates and allowed to grow to 90–95% confluence. Six hours after transfection, a single scratch wound was generated in each well. The cells were washed with PBS supplemented with serum-free medium to remove cell fragments and then monitored. Images were captured by phase-contrast microscopy at 0, 24, and 48 h after wounding.

### RNA Extraction, Reverse Transcription, and Quantitative Real-Time PCR (qRT-PCR)

Total RNA was extracted from cultured cells using TRIzol reagent (Invitrogen, Thermo Fisher Scientific) according to the manufacturer's protocols. cDNA was synthesized from one microgram of total RNA by using a Reverse Transcription Kit (Applied Biosystems, Thermo Fisher Scientific). qRT-PCR was performed using PowerUp^TM^ SYBR Green Master Mix (Thermo Scientific, Waltham, USA). The relative expression of PCAT6 and TAK1 was normalized to β-actin expression. The primer sequences are listed in Supplementary Table 1. For analysis of the expression of miR-143-3p, Bulge-loop^TM^ miRNA qRT-PCR Primer Sets specific for miR-143-3p were designed by RiboBio (Guangzhou, China). miR-143-3p was quantified with a stem-loop real-time PCR miRNA kit (RiboBio, Guangzhou, China). The mRNA levels were normalized to U6 snRNA levels.

### Small-RNA Sequencing and Bioinformatic Analysis

To explore miRNAs that bind to PCAT6, we performed small-RNA sequencing in A2780 cells after transfection with PCAT6-specific siRNAs (Si-PCAT6) and an NC siRNA (Si-NC). Briefly, total RNA was extracted with TRIzol, and RNA molecules in a size range of 18–30 nt were enriched by polyacrylamide gel electrophoresis (PAGE). Then, 3′ adapters were added, the 36–44 nt RNAs were enriched, and 5′ adapters were ligated to the RNAs. The ligation products were reverse transcribed by PCR amplification, and the 140–160 bp PCR products were enriched to generate a cDNA library, which was sequenced using an Illumina HiSeq^TM^ 2500 (Zeng et al., 2019). Based on the expression in each sample, the miRNA expression level was calculated and normalized to the transcripts per million (TPM) value. Differentially expressed (DE) miRNAs were identified by the edgeR package in the R program. We considered miRNAs with a fold change values ≥1 and *P*-values < 0.05 as significant DE miRNAs. Then, the Encyclopedia of RNA Interactomes (ENCORI, http://starbase.sysu.edu.cn/) was used to explore the miRNAs that target PCAT6. Furthermore, we used the GEO2R (http://www.ncbi.nlm.nih.gov/geo/geo2r/) tool to analyse the expression patterns of the candidate miRNAs in GEO miRNA datasets of ovarian cancer patients, namely, GSE83693, GSE119055, and GSE131790.

### Luciferase Assay

The wild-type (WT) or mutant (MUT) PCAT6-binding sites were subcloned into the pmiR-RB-Report^TM^ vector (RiboBio, Guangzhou, China). HEK293T cells were seeded onto 96-well plates. miR-143-3p mimics or the NC sequence (RiboBio, Guangzhou, China) were co-transfected with pmiR-RB-Report-WT-PCAT6 or pmiR-RB-Report-MUT-PCAT6. Two days after transfection, the cells were collected, and a luciferase activity was performed using a Dual Luciferase Reporter Assay System (Promega, Madison, WI, USA).

### Western Blot Assay

After transient transfection in 6-well plates for 48 h, 80–100 μl/well of protein lysate (RIPA: PMSF = 100:1) was added to the 6-well plates and incubated on ice for a few minutes. After the cells were fully lysed, the proteins were scraped off using a spatula, transferred to 1.5 ml tubes, and centrifuged at 4°C and 12,000 rpm for 15–20 min. The supernatant was aspirated. Protein-loading buffer (G2013, Servicebio, Wuhan, China) was added, and the samples were incubated for 10 min at 95°C. The protein molecules were separated by SDS-PAGE, which was stopped when the target bands migrated to the appropriate positions for wet transfer. The proteins were transferred to a film in an ice bath at 350–400 mA for 90 min. The PVDF membrane was placed in 5% skimmed milk and blocked at room temperature for 2 h in a horizontal shaker. The blots were probed overnight at 4°C with primary antibodies: a rabbit anti-TAK1 antibody (Abcam, Cambridge, MA, USA, 1:1,000, ab109526) and a mouse anti-β-actin antibody (Proteintech, Wuhan, China, 1:1,000, 66009-I-Ig). After incubation with primary antibodies, the PVDF membrane was washed 3 times with TBST and incubated with goat anti-rabbit IgG (Biosharp, Anhui, China, 1: 20,000, BL003A) and goat anti-mouse IgG (Biosharp, Anhui, China, 1: 20,000, BL001A) secondary antibodies for 2 h. Images were obtained using a FluorChem M Multicolor fluorescence and chemiluminescence gel imaging system (ProteinSimple, Silicon Valley, USA).

### Statistical Analysis

All data are presented as the mean ± standard deviation. Unless otherwise noted, the significance of differences between groups was estimated using Student's *t*-test. A value of *P* < 0.05 indicated a significant difference. All statistical analyses were performed using SPSS 20.0 software (IBM, Chicago, IL, USA).

## Results

### PCAT6 Is Significantly Upregulated in Ovarian Cancer Tissues and Predicts Poor Prognosis of Ovarian Cancer Patients

**Figure 1:**
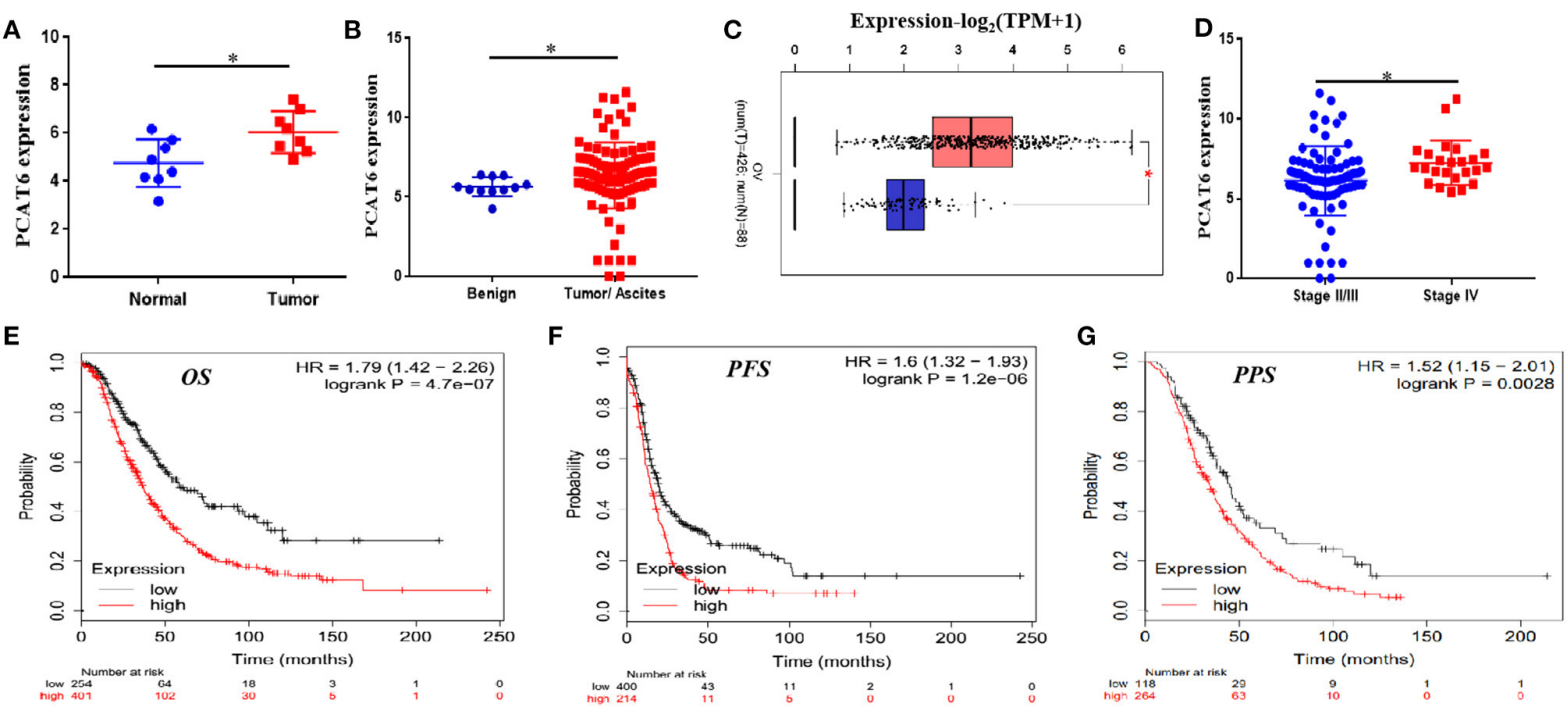
PCAT6 is overexpressed in ovarian cancer tissues and correlates with poor prognosis of ovarian cancer patients. **(A)** Analysis of PCAT6 expression in primary ovarian cancer patient samples and matched normal fallopian tube samples based on GSE137238 data. **(B)** Analysis of PCAT6 expression in serous ovarian cancer tissues or ascites and benign tissues based on GSE143897 data. **(C)** Analysis of PCAT6 expression in The Cancer Genome Atlas (TCGA) ovarian cancer tissues and Genotype-Tissue Expression (GTEx) normal ovary tissues using the GEPIA web server. **(D)** Levels of PCAT6 expression in patients with different stages of ovarian cancer based on the GSE143897 dataset. **(E–G)** Kaplan–Meier Plotter results for OS, PFS, and PPS of ovarian cancer patients with low and high PCAT6 expression. *P < 0.05.

In the GSE137238 dataset, we first observed significant upregulation of PCAT6 expression in primary ovarian cancer patient samples compared with matched normal fallopian tube samples (*P* = 0.015, Figure 1A). Then, we found that PCAT6 levels were higher in serous ovarian cancer tissues and ascites than in benign tissues based on the GSE143897 dataset (*P* = 0.009, Figure 1B). We verified the expression of PCAT6 in ovarian cancer and normal ovarian tissues using GEPIA and confirmed that PCAT6 was significantly overexpressed in ovarian cancer tissues (*P* < 0.05, Figure 1C). Furthermore, among patients with different stages of ovarian cancer based on the GSE143897 dataset, the expression of PCAT6 significantly increased from stage II/III to stage IV (*P* = 0.02, Figure 1D). The prognostic value of PCAT6 in ovarian cancer patients was assessed, and Kaplan–Meier Plotter analysis demonstrated that high expression of PCAT6 was correlated with poor OS, PFS and PPS in ovarian cancer patients (Figures 1E–G, all *P* < 0.05). The above findings indicate that PCAT6 may play oncogenic roles in the development and progression of ovarian cancer.

### PCAT6 Promotes Malignant Phenotypes of Ovarian Cancer *in vitro*

**Figure 2:**
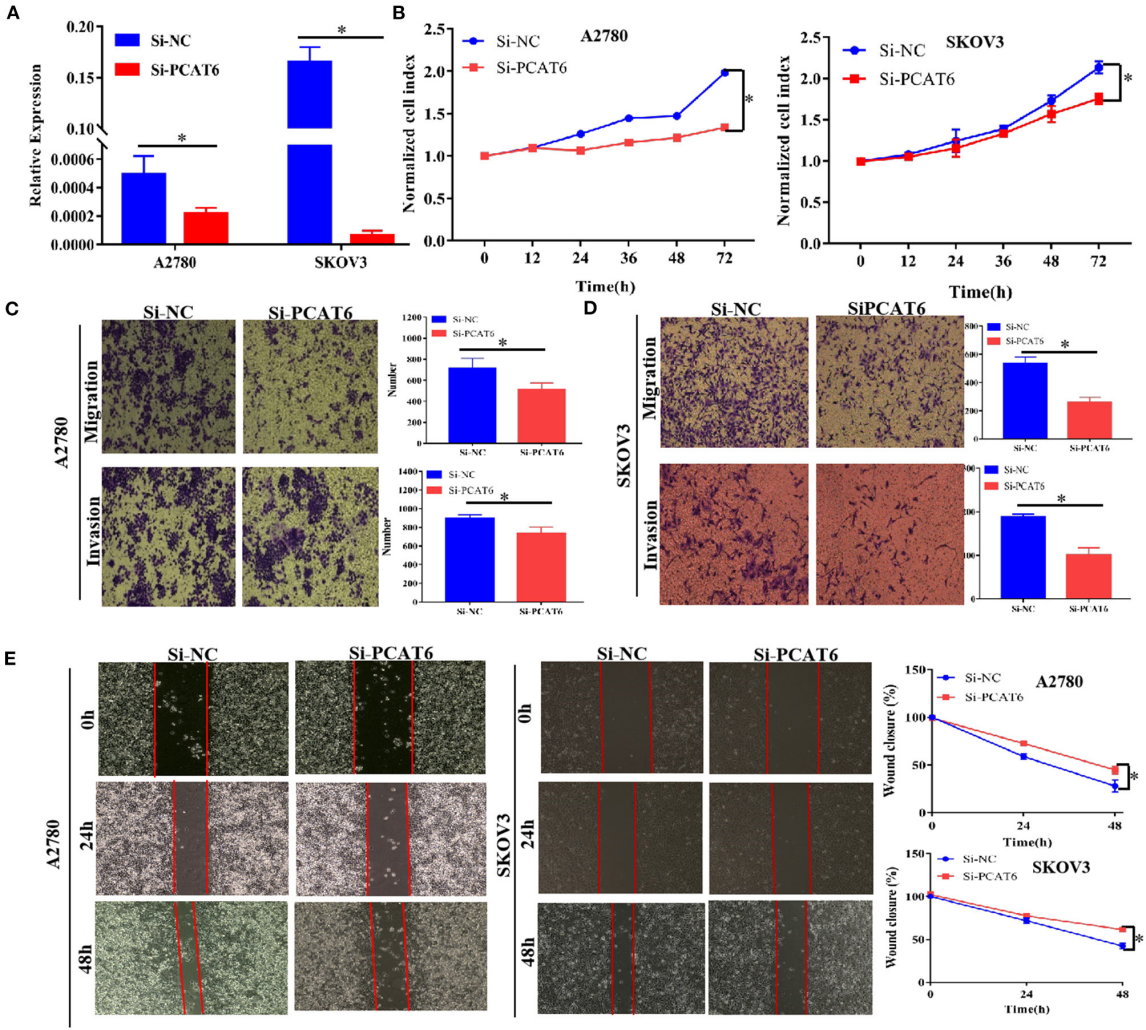
Knockdown of PCAT6 in A2780 and SKOV3 cells inhibits ovarian cancer cell proliferation, migration and invasion. **(A)** The expression of PCAT6 was significantly lower in Si-PCAT6-transfected A2780 and SKOV3 cells than in Si-NC-transfected (control) cells. **(B)** CCK-8 assays showed that knockdown of PCAT6 inhibited A2780 (left) and SKOV3 (right) cell proliferation. **(C)** Representative images of the results of Transwell migration and invasion assays in A2780 cells (left). The mean numbers of migrated and invaded cells were lower in the Si-PCAT6 group than in the Si-NC group (right). **(D)** Representative images of the results of Transwell migration and invasion assays in SKOV3 cells (left). The mean numbers of migrated and invaded cells were lower in the Si-PCAT6 group than in the Si-NC group (right). **(E)** Representative images of wound healing assays in A2780 and SKOV3 cells (left). The motility of ovarian cancer cells was lower in the Si-PCAT6 group than in the Si-NC group (right). *P < 0.05.

To better understand the function of PCAT6 in ovarian cancer development, we then examined whether knockdown or overexpression of PCAT6 affects the proliferation, migration and invasion of ovarian cancer cells. We first used Si-PCAT6 to inhibit endogenous PCAT6 expression in SKOV3 and A2780 cells. In both cell types, PCAT6 knockdown resulted in lower PCAT6 expression than that in the control group (Figure 2A). Then, we found that PCAT6 knockdown significantly suppressed the viability of A2780 and SKOV3 cells by CCK-8 assay (Figure 2B). In migration and invasion assays, the numbers of migrated and invaded A2780 and SKOV3 cells in the Si-PCAT6 group were markedly lower than those in the NC group (*P* < 0.05, Figures 2C,D). The wound healing assay also showed that PCAT6 knockdown suppressed the migration of A2780 and SKOV3 cells (Figure 2E). Additionally, we overexpressed PCAT6 by transfecting pcDNA3.1-PCAT6 into A2780 and SKOV3 cells. The level of PCAT6 in the overexpression group was markedly higher than that in the control group in both cell lines (Supplementary Figure 1A). Although overexpression of PCAT6 induced cell proliferation (Supplementary Figure 1B), the differences between the PCAT6-overexpressing cells and the control cells were small. Consistently, overexpression of PCAT6 increased the numbers of migrated and invaded cells (Supplementary Figures 1C,D) and promoted migration (Supplementary Figure 1E).

### The lncRNA PCAT6 Acts as a ceRNA to Regulate TAK1 Expression by Binding to miR-143-3p

**Figure 3:**
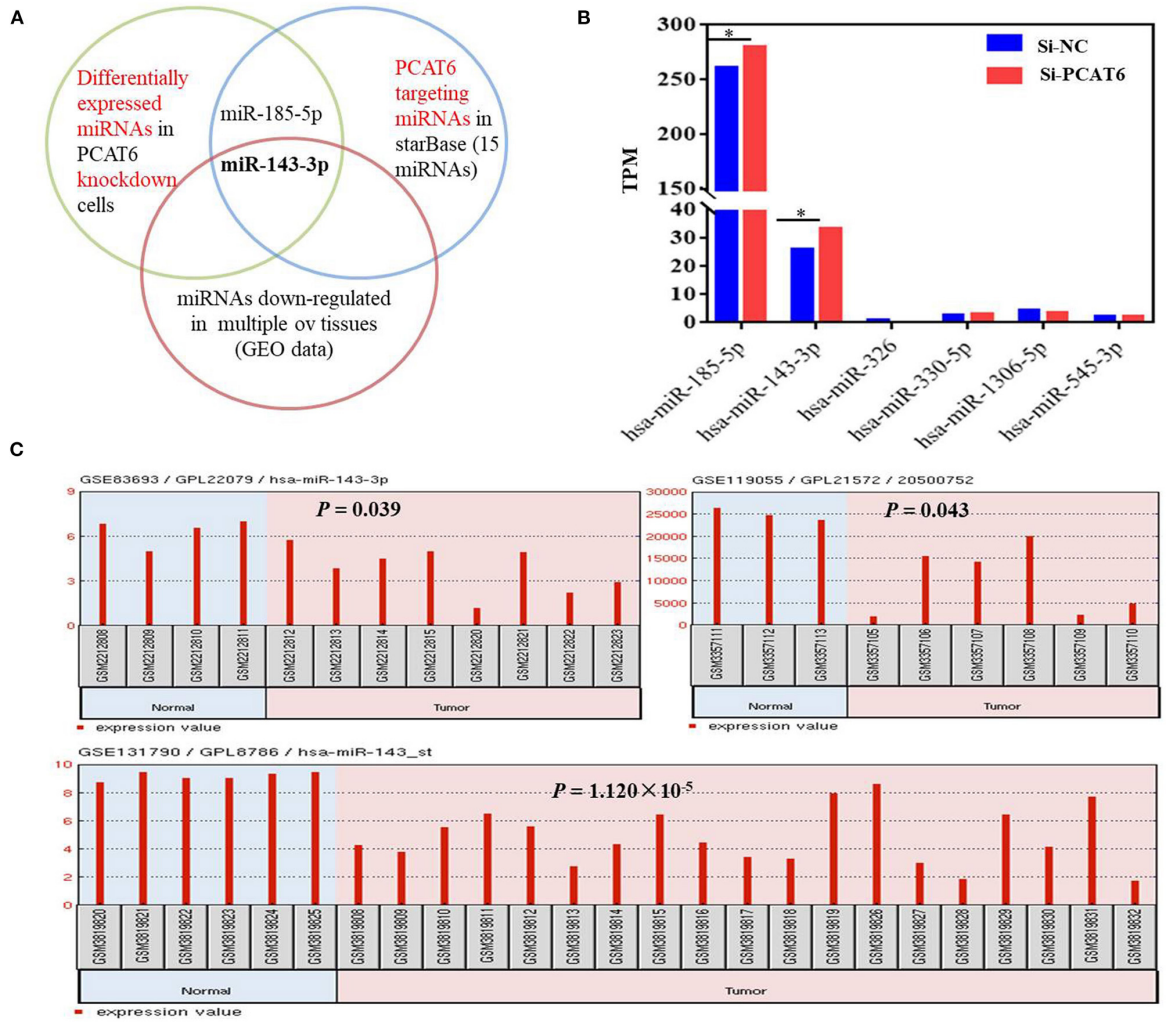
miR-143-3p is a potential miRNA target of PCAT6 in ovarian cancer. **(A)** Diagram showing the intersection among DE miRNAs in Si-PCAT6-transfected A2780 cells compared with Si-NC-transfected A2780 cells (determined using small-RNA sequencing); PCAT6-targeting miRNAs from the ENCORI database; and miRNAs that were significantly downregulated in multiple ovarian cancer tissues based on the GSE83693, GSE119055, and GSE131790 datasets. **(B)** The expression levels of six miRNAs cataloged in the ENCORI database were detected in our small-RNA sequencing data. **(C)** miR-143-3p was consistently and significantly downregulated in tumor tissues compared to normal tissues based on the GSE83693, GSE119055, and GSE131790 datasets. *P < 0.05.

**Figure 4:**
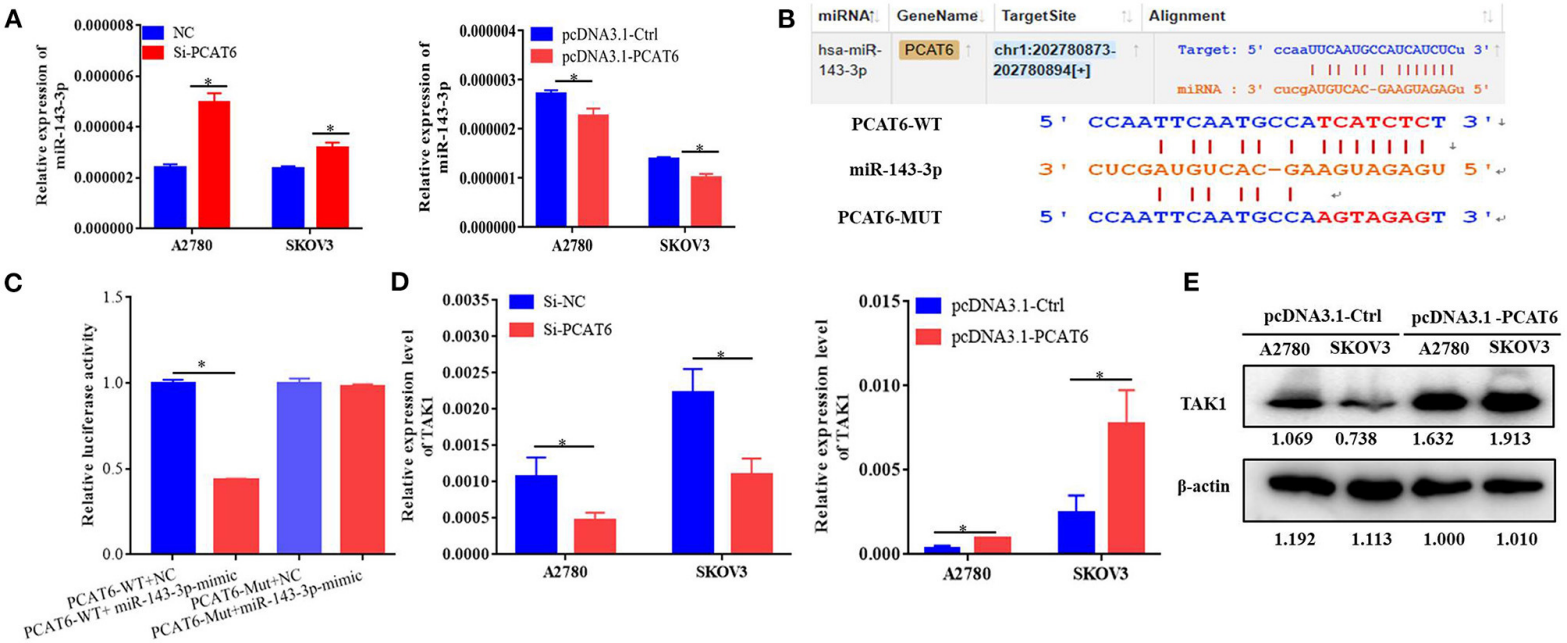
PCAT6 competitively binds to miR-143-3p and regulates TAK1 expression. **(A)** Expression of miR-143-3p in A2780 and SKOV3 cells treated with Si-PCAT6 or PCDNA3.1-PCAT6. **(B)** Binding sites of PCAT6 and miR-143-3p identified by the online website ENCORI. **(C)** The binding of PCAT6 and miR-143-3p was verified by dual-luciferase reporter assay. The relative luciferase reporter activity was reduced in cells co-transfected with miR-143-3p and a WT PCAT6 reporter construct. **(D)** Expression of TAK1 in A2780 and SKOV3 cells treated with Si-PCAT6 or PCDNA3.1-PCAT6. **(E)** Protein expression of TAK1 in A2780 and SKOV3 cells treated with PCDNA3.1-PCAT6. *P < 0.05.

To identify the potential miRNA targets of lncRNA PCAT6, we performed small-RNA sequencing in A2780 cells transfected with siRNA or an NC sequence. We identified 390 DE miRNAs (Supplementary Table 2). Then, *in silico* analysis was performed by using ENCORI databases, which predicted that 15 miRNAs bind to PCAT6 (Supplementary Table 3, Figure 3A). The expression levels of six miRNAs were detected in our small-RNA sequencing data (Figure 3B), but only two miRNAs (miR-143-3p and miR-185-5p) were upregulated after PCAT6 was inhibited. miR-143-3p expression was consistently downregulated in ovarian cancer samples compared to normal tissues in the GSE83693, GSE119055, and GSE131790 datasets (Figure 3C), while miR-185-5p expression was upregulated in the GSE83693 and GSE119055 datasets but downregulated in the GSE131790 dataset (Supplementary Figure 2). Additionally, the expression of miR-143-3p was increased after PCAT6 was inhibited, while it was decreased after PCAT6 was overexpressed in both SKOV3 and A2780 ovarian cancer cells (Figure 4A). We further validated the binding of miR-143-3p with PCAT6 (Figure 4B) by using a luciferase reporter assay. As shown in Figure 4C, the miR-143-3p mimic markedly decreased the luciferase activity of PCAT6-WT but not PCAT6-MUT in HEK293T cells, indicating that miR-143-3p is a target of the lncRNA PCAT6. Based on these findings, we searched published studies and found that TAK1 has been identified as a direct target of miR-143-3p in ovarian cancer (Shi H. et al., 2018). In our study, TAK1 mRNA expression was significantly decreased when PCAT6 was inhibited but increased when PCAT6 was overexpressed in both cell lines (Figure 4D). We confirmed that TAK1 protein expression was upregulated in PCAT6-overexpressing cells (Figure 4E). Furthermore, we explored the role of TAK1 in PCAT6-overexpressing cells. Cell proliferation and invasion assays illustrated that silencing TAK1 in A2780 and SKOV3 cells inhibited the malignant phenotype of PCAT6 overexpression (Supplementary Figure 3). All these findings indicate that PCAT6 might affect the behaviors of ovarian cancer cells by regulating TAK1.

### miR-143-3p Reverses the Function of PCAT6

**Figure 5:**
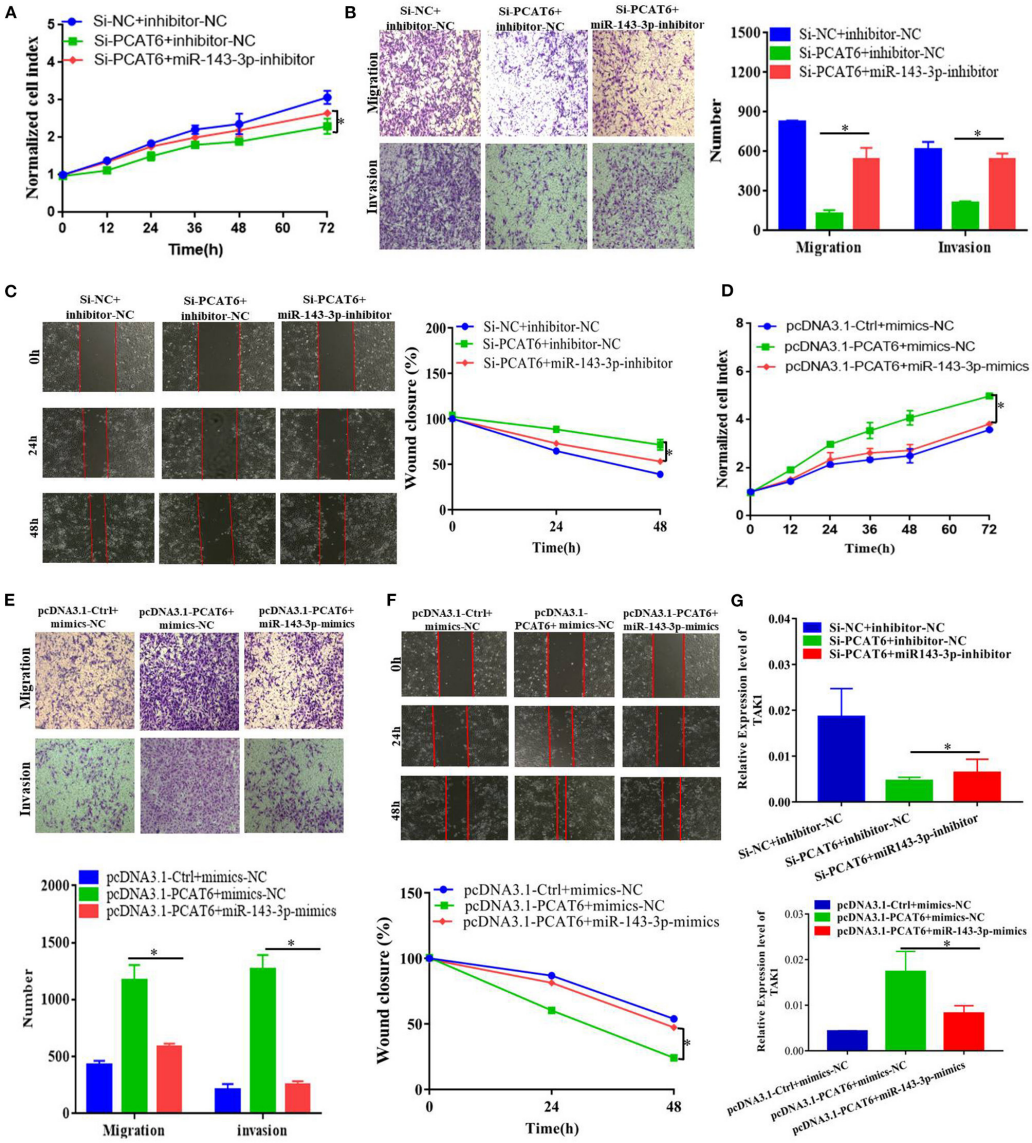
PCAT6 affects ovarian cancer cell proliferation, migration, and invasion by modulating miR-143-3p. **(A)** Downregulation of miR-143-3p rescued the proliferation of SKOV3 cells with knockdown of PCAT6. **(B)** Downregulation of miR-143-3p rescued the migration and invasion abilities of SKOV3 cells with knockdown of PCAT6. **(C)** Downregulation of miR-143-3p rescued the migration of SKOV3 cells with knockdown of PCAT6, as demonstrated by wound healing assay. **(D)** Upregulation of miR-143-3p rescued the proliferation of SKOV3 cells overexpressing PCAT6. **(E)** Upregulation of miR-143-3p rescued the migration and invasion abilities of SKOV3 cells overexpressing PCAT6. **(F)** Upregulation of miR-143-3p rescued the migration of SKOV3 cells overexpressing PCAT6, as demonstrated by wound healing assay. **(G)** miR-143-3p rescued the expression of TAK1 in SKOV3 cells co-transfected with Si-PCAT6 and a miR-143-3p inhibitor and in PCAT6-overexpressing SKOV3 cells transfected with miR-143-3p mimics. *P < 0.05.

Considering that PCAT6 acts as a ceRNA to regulate TAK1 expression by binding to miR-143-3p, we performed rescue assays to validate whether miR-143-3p is involved in the PCAT6-mediated promotion of proliferation, migration, and invasion in ovarian cancer cells. In SKOV3 cells, we observed that downregulation of miR-143-3p mitigated PCAT6 knockdown-induced suppression of proliferation, migration, and invasion (Figures 5A–C). On the other hand, upregulation of miR-143-3p reversed the PCAT6 overexpression-induced enhancement of proliferation, migration, and invasion (Figures 5D–F). Moreover, we observed that miR-143-3p rescued the expression of TAK1 in SKOV3 cells co-transfected with Si-PCAT6 and a miR-143-3p inhibitor and in PCAT6-overexpressing SKOV3 cells transfected with miR-143-3p mimics (Figure 5G).

## Discussion

Since lncRNAs play vital regulatory roles in tumorigenesis and progression, much attention has been given to the functions and mechanisms of lncRNAs in recent years. In the present study, we found that PCAT6 was upregulated in ovarian cancer tissues and that elevated expression of PCAT6 was correlated with reduced OS, PFS and PPS in ovarian cancer patients. Functional experiments demonstrated that PCAT6 promoted ovarian cancer cell proliferation, migration and invasion. Moreover, PCAT6 promoted malignancy of ovarian cancer via its interaction with miR-143-3p and effects on TAK1 expression. Taken together, these findings indicate that the lncRNA PCAT6 plays an oncogenic role in the development of ovarian cancer.

The lncRNA PCAT6 is a cancer/testis (CT) lncRNA that is normally silenced in healthy tissues except for the testes but is highly expressed in malignancies (Wang et al., 2016; Chen et al., 2019). Emerging evidence has shown that PCAT6 expression is upregulated in various cancers and plays oncogenic roles in cancer development. For instance, Chen et al. demonstrated that PCAT6 is significantly upregulated in hepatocellular carcinoma (HCC) tissues and that knockdown of PCAT6 in HCC cells can inhibit cell growth and migration (Chen et al., 2019). Luo et al. further confirmed that PCAT6 can predict poor prognosis in HCC and promote proliferation through regulation of cell cycle arrest and apoptosis (Luo et al., 2020). Based on pathway crosstalk analysis, PCAT6 has been identified as a hub lncRNA that is closely related to the clinical features of lung adenocarcinoma (Qi et al., 2019). Several studies have consistently demonstrated that aberrant upregulation of PCAT6 is common in lung cancer tissues and promotes the proliferation, migration, and invasion of lung cancer cells (Wan et al., 2016; Cui et al., 2018; Shi X. et al., 2018). PCAT6 has also been reported to be overexpressed in gastric cancer tissues and to promote the development of gastric cancer (Xu et al., 2018). Ma et al. revealed that the levels of PCAT6 are enhanced in cervical cancer (CC) tissues and that PCAT6 accelerates the proliferation and metastasis of CC cells while suppressing apoptosis (Ma et al., 2020). More recently, findings by Kong et al. have indicated that PCAT6 is highly expressed in ovarian cancer tissues and cell lines and that knockdown of PCAT6 inhibits cell proliferation, invasion and migration in the SKOV3 and CAOV3 cell lines (Kong et al., 2019). Despite these findings, little is known about the underlying mechanisms of PCAT6 in ovarian cancer carcinogenesis. The key findings of our present study are that PCAT6 is significantly elevated in ovarian cancer tissues and that this elevation is associated with advanced TNM stage and poor prognosis, indicating that PCAT6 is a potential diagnostic and prognostic marker for ovarian cancer. Further functional experiments in two cell lines demonstrated that knockdown of PCAT6 suppresses cell proliferation, migration and invasion, while overexpression of PCAT6 produces the opposite results, indicating that PCAT6 exerts an oncogenic function in ovarian cancer development.

Accumulating evidence has shown that lncRNAs function as ceRNAs for specific miRNAs to regulate the target genes of these miRNAs (Liang et al., 2018; Gokulnath et al., 2019; Qi et al., 2020). Until now, the details of the underlying mechanism of PCAT6 in ovarian cancer have remained elusive. To explore whether PCAT6 can act as a ceRNA in ovarian cancer, we combined small-RNA sequencing, bioinformatic analysis and dual-luciferase reporter assays and verified that miR-143-3p was a direct target of PCAT6. miR-143-3p was negatively regulated by PCAT6 in ovarian cancer cells. The PCAT6 overexpression-induced increases in cell growth and metastasis were attenuated by the miR-143-3p mimic, while PCAT6 knockdown-induced suppression was partly rescued. Therefore, we propose that PCAT6 may play a major role in ovarian cancer cells by sponging miR-143-3p during ovarian cancer progression. Recent reports have demonstrated that miR-143-3p acts as a tumor suppressor in multiple kinds of malignant cancers, such as HCC, esophageal squamous cell carcinoma, bladder cancer and ovarian cancer (Noguchi et al., 2011; He et al., 2016; Shi H. et al., 2018; Peng et al., 2020). Interestingly, it has been reported that miR-143-3p regulates the expression of TAK1 in ovarian cancer cells and exerts an inhibitory effect on the proliferation, migration, and invasion of ovarian cancer cells (Shi H. et al., 2018). A direct interaction between TAK1 and miR-143 has been further demonstrated in pancreatic ductal adenocarcinoma (Huang et al., 2017). In our study, we observed that the expression of PCAT6 was significantly repressed in PCAT6-knockdown cells and upregulated in PCAT6-overexpressing cells. According to previous studies, TAK1 is a serine/threonine kinase of the MAP3K family (MAP3K7) that is a critical regulator of TGF-β signaling and participates in the activation of p38MAPK and JNK in various cellular systems (Safina et al., 2008; Rincón and Davis, 2009). Importantly, it has been reported that elevated TAK1 levels promote ovarian cancer cell growth and metastatic capacity through activation of NF-κB signaling (Cai et al., 2014). Based on these results, we infer that PCAT6 plays oncogenic roles in the malignancy of ovarian cancer by sponging miR-143-3p and reducing its activity, thus increasing TAK1 expression.

Notably, there are several limitations of our study. First, the SKOV3 and A2780 cell lines were used for both PCAT6 overexpression and knockdown experiments to evaluate the malignant phenotype of ovarian cancer. More representative cell lines, particularly stably lentivirus-transfected cell lines, should be used to validate our results. Second, we did not study the function or mechanism of PCAT6 *in vivo*. In future studies, we will evaluate the roles of PCAT6 *in vivo*. In summary, our study establishes the clinical and oncogenic functions of PCAT6 in ovarian tumorigenesis and progression. Additionally, we demonstrate that the oncogenic activity of PCAT6 is attributable to the ceRNA regulatory network of the PCAT6-miR-143-3p-TAK1 axis, which is a potential therapeutic target for ovarian cancer.

## Data Availability Statement

The raw data supporting the conclusions of this article will be made available by the authors, without undue reservation.

## Author Contributions

KX, HX, and JH designed the study. XT, YS, YT, SL, and WL performed the experiments. LX, YC, CS, JZ, and JH analyzed the public database and discussed the results. KX, XT, and YT prepared the manuscript, and all authors approved the final manuscript.

## Conflict of Interest

The authors declare that the research was conducted in the absence of any commercial or financial relationships that could be construed as a potential conflict of interest.
