# Supplementary material for: The Cancer-Testis Long Non-coding RNA PCAT6 Facilitates the Malignant Phenotype of Ovarian Cancer by Sponging miR-143-3p
Source: Front Cell Dev Biol. 2021 Feb 4;9:593677. doi: 10.3389/fcell.2021.593677 (PMC7902004; doi:10.3389/fcell.2021.593677)
Supplement: Supplementary file 1 [file Table_1.DOC]

| Supplementary Table 1. Primer sequences for RT-PCR | | |
| --- | --- | --- |
| Gene | Forward (5'-3') | Reverse (5'-3') |
| PCAT6 | AGCCTTCGCCCCTAGATACA | TGGTAGAAGCACGAGCAAGG |
| TAK1 | CAAAGCTAAGTGGAGAGCAAAAGA | GATAACTGCCGAAGCTCTACAATAA |
| β-actin | AGTACTCCGTGTGGATCGGC | GCTGATCCACATCTGCTGGA |

| Supplementary Table 2. Differentially expressed miRNAs between Si-NC and Si-PCAT6 groups | | | | |
| --- | --- | --- | --- | --- |
| miRNA name | NC_TPM | SiPCAT6_TPM | FC | *P* value |
| hsa-let-7a-3p | 44.218 | 49.156 | 1.112 | 0.022 |
| hsa-miR-100-5p | 73148.693 | 76708.098 | 1.049 | 0.000 |
| hsa-miR-101-3p | 607.756 | 648.392 | 1.067 | 0.000 |
| hsa-miR-103a-3p | 3962.550 | 4685.191 | 1.182 | 0.000 |
| hsa-miR-105-3p | 0.010 | 1.902 | 190.190 | 0.001 |
| hsa-miR-10a-3p | 380.418 | 381.984 | 1.004 | 0.001 |
| hsa-miR-10a-5p | 43913.199 | 59910.755 | 1.364 | 0.000 |
| hsa-miR-10b-5p | 6502.678 | 8494.496 | 1.306 | 0.000 |
| hsa-miR-122-5p | 2.163 | 7.169 | 3.314 | 0.000 |
| hsa-miR-1255a | 1.202 | 3.804 | 3.166 | 0.008 |
| hsa-miR-1255b-5p | 0.481 | 2.780 | 5.784 | 0.004 |
| hsa-miR-126-3p | 99.971 | 102.847 | 1.029 | 0.034 |
| hsa-miR-1273c | 3.364 | 6.437 | 1.913 | 0.015 |
| hsa-miR-1283 | 0.721 | 5.120 | 7.103 | 0.000 |
| hsa-miR-1285-5p | 0.481 | 2.048 | 4.262 | 0.035 |
| hsa-miR-1301-3p | 21.148 | 27.943 | 1.321 | 0.002 |
| hsa-miR-130a-5p | 0.961 | 3.657 | 3.805 | 0.006 |
| hsa-miR-135b-5p | 13.458 | 16.971 | 1.261 | 0.037 |
| hsa-miR-137-3p | 37.249 | 47.986 | 1.288 | 0.000 |
| hsa-miR-137-5p | 0.010 | 1.170 | 117.040 | 0.016 |
| hsa-miR-138-5p | 18.024 | 26.773 | 1.485 | 0.000 |
| hsa-miR-140-5p | 15.140 | 33.649 | 2.223 | 0.000 |
| hsa-miR-143-3p | 25.714 | 33.210 | 1.292 | 0.002 |
| hsa-miR-146b-5p | 566.422 | 904.999 | 1.598 | 0.000 |
| hsa-miR-148a-3p | 918.964 | 934.112 | 1.016 | 0.000 |
| hsa-miR-148b-5p | 15.140 | 19.750 | 1.305 | 0.014 |
| hsa-miR-151a-3p | 1093.673 | 1528.228 | 1.397 | 0.000 |
| hsa-miR-155-5p | 55.272 | 85.145 | 1.540 | 0.000 |
| hsa-miR-15a-5p | 124.002 | 153.174 | 1.235 | 0.000 |
| hsa-miR-15b-3p | 96.847 | 118.355 | 1.222 | 0.000 |
| hsa-miR-15b-5p | 1656.730 | 1667.943 | 1.007 | 0.000 |
| hsa-miR-16-2-3p | 138.902 | 139.276 | 1.003 | 0.041 |
| hsa-miR-17-3p | 50.226 | 54.715 | 1.089 | 0.025 |
| hsa-miR-17-5p | 508.506 | 557.249 | 1.096 | 0.000 |
| hsa-miR-181a-3p | 860.808 | 865.499 | 1.005 | 0.000 |
| hsa-miR-181a-5p | 2489.180 | 5850.600 | 2.350 | 0.000 |
| hsa-miR-181b-2-3p | 4.806 | 7.461 | 1.552 | 0.039 |
| hsa-miR-181b-5p | 1234.978 | 1433.135 | 1.160 | 0.000 |
| hsa-miR-181c-5p | 12.256 | 23.115 | 1.886 | 0.000 |
| hsa-miR-181d-5p | 44.458 | 53.106 | 1.195 | 0.002 |
| hsa-miR-182-5p | 674.323 | 1054.223 | 1.563 | 0.000 |
| hsa-miR-185-5p | 261.222 | 279.429 | 1.070 | 0.000 |
| hsa-miR-186-5p | 125.204 | 176.874 | 1.413 | 0.000 |
| hsa-miR-190a-3p | 7.690 | 15.361 | 1.998 | 0.000 |
| hsa-miR-191-5p | 6300.332 | 7018.350 | 1.114 | 0.000 |
| hsa-miR-193b-5p | 37.249 | 54.715 | 1.469 | 0.000 |
| hsa-miR-196b-5p | 13.217 | 16.971 | 1.284 | 0.030 |
| hsa-miR-197-5p | 0.010 | 1.024 | 102.410 | 0.031 |
| hsa-miR-199a-3p | 9.372 | 18.726 | 1.998 | 0.000 |
| hsa-miR-199a-5p | 10.574 | 19.604 | 1.854 | 0.000 |
| hsa-miR-199b-3p | 9.372 | 18.726 | 1.998 | 0.000 |
| hsa-miR-199b-5p | 0.721 | 3.365 | 4.668 | 0.002 |
| hsa-miR-19a-3p | 7.450 | 44.767 | 6.009 | 0.000 |
| hsa-miR-19b-3p | 48.784 | 130.352 | 2.672 | 0.000 |
| hsa-miR-20a-3p | 1.442 | 3.804 | 2.638 | 0.016 |
| hsa-miR-20a-5p | 1325.576 | 1722.219 | 1.299 | 0.000 |
| hsa-miR-2110 | 3.364 | 6.730 | 2.000 | 0.008 |
| hsa-miR-21-5p | 233378.328 | 257621.573 | 1.104 | 0.000 |
| hsa-miR-219a-5p | 2.644 | 5.267 | 1.992 | 0.023 |
| hsa-miR-221-3p | 2233.486 | 2453.563 | 1.099 | 0.000 |
| hsa-miR-221-5p | 301.595 | 423.386 | 1.404 | 0.000 |
| hsa-miR-222-3p | 649.571 | 817.074 | 1.258 | 0.000 |
| hsa-miR-224-5p | 793.279 | 1223.343 | 1.542 | 0.000 |
| hsa-miR-2355-3p | 9.372 | 13.313 | 1.420 | 0.020 |
| hsa-miR-23a-3p | 12179.874 | 12481.993 | 1.025 | 0.000 |
| hsa-miR-23a-5p | 15.861 | 22.384 | 1.411 | 0.002 |
| hsa-miR-24-2-5p | 184.802 | 219.886 | 1.190 | 0.000 |
| hsa-miR-26b-5p | 629.384 | 661.706 | 1.051 | 0.000 |
| hsa-miR-27a-5p | 1029.749 | 2204.710 | 2.141 | 0.000 |
| hsa-miR-27b-5p | 83.149 | 105.335 | 1.267 | 0.000 |
| hsa-miR-29c-3p | 16.341 | 20.628 | 1.262 | 0.021 |
| hsa-miR-301a-3p | 1.442 | 3.950 | 2.739 | 0.011 |
| hsa-miR-30a-3p | 1899.688 | 2248.892 | 1.184 | 0.000 |
| hsa-miR-30a-5p | 23821.442 | 33094.492 | 1.389 | 0.000 |
| hsa-miR-30b-5p | 583.244 | 647.807 | 1.111 | 0.000 |
| hsa-miR-30c-2-3p | 290.781 | 300.057 | 1.032 | 0.000 |
| hsa-miR-30c-5p | 2806.636 | 4417.027 | 1.574 | 0.000 |
| hsa-miR-30d-5p | 1449.579 | 1802.537 | 1.243 | 0.000 |
| hsa-miR-30e-3p | 75.699 | 90.120 | 1.190 | 0.000 |
| hsa-miR-30e-5p | 1272.947 | 1828.139 | 1.436 | 0.000 |
| hsa-miR-3129-5p | 0.010 | 2.780 | 277.970 | 0.000 |
| hsa-miR-31-3p | 212.438 | 243.586 | 1.147 | 0.000 |
| hsa-miR-3144-5p | 0.481 | 2.780 | 5.784 | 0.004 |
| hsa-miR-3180 | 0.010 | 1.024 | 102.410 | 0.031 |
| hsa-miR-320a-5p | 1.202 | 4.682 | 3.896 | 0.001 |
| hsa-miR-330-3p | 16.341 | 21.798 | 1.334 | 0.009 |
| hsa-miR-339-3p | 36.528 | 48.717 | 1.334 | 0.000 |
| hsa-miR-342-5p | 0.010 | 3.950 | 395.000 | 0.000 |
| hsa-miR-34c-5p | 49.505 | 55.886 | 1.129 | 0.008 |
| hsa-miR-361-3p | 26.915 | 44.767 | 1.663 | 0.000 |
| hsa-miR-361-5p | 761.077 | 773.331 | 1.016 | 0.000 |
| hsa-miR-362-5p | 13.458 | 39.208 | 2.913 | 0.000 |
| hsa-miR-365a-5p | 8.411 | 13.459 | 1.600 | 0.004 |
| hsa-miR-374a-3p | 124.243 | 132.839 | 1.069 | 0.002 |
| hsa-miR-374b-5p | 205.229 | 221.641 | 1.080 | 0.000 |
| hsa-miR-3913-5p | 0.961 | 2.633 | 2.739 | 0.041 |
| hsa-miR-411-3p | 0.010 | 1.463 | 146.300 | 0.004 |
| hsa-miR-423-5p | 607.996 | 723.736 | 1.190 | 0.000 |
| hsa-miR-424-5p | 66.087 | 90.120 | 1.364 | 0.000 |
| hsa-miR-425-5p | 66.327 | 138.398 | 2.087 | 0.000 |
| hsa-miR-429 | 57.676 | 84.121 | 1.459 | 0.000 |
| hsa-miR-4424 | 0.010 | 2.195 | 219.450 | 0.000 |
| hsa-miR-450a-1-3p | 0.010 | 1.609 | 160.930 | 0.004 |
| hsa-miR-450a-5p | 183.360 | 235.979 | 1.287 | 0.000 |
| hsa-miR-4521 | 21.388 | 51.790 | 2.421 | 0.000 |
| hsa-miR-452-5p | 86.754 | 207.012 | 2.386 | 0.000 |
| hsa-miR-454-3p | 3.605 | 11.558 | 3.206 | 0.000 |
| hsa-miR-455-5p | 166.538 | 198.965 | 1.195 | 0.000 |
| hsa-miR-4672 | 0.010 | 1.170 | 117.040 | 0.016 |
| hsa-miR-4728-5p | 0.010 | 1.317 | 131.670 | 0.008 |
| hsa-miR-4755-3p | 0.481 | 2.195 | 4.566 | 0.021 |
| hsa-miR-4761-3p | 0.010 | 1.024 | 102.410 | 0.031 |
| hsa-miR-4775 | 0.481 | 3.219 | 6.697 | 0.001 |
| hsa-miR-491-5p | 0.961 | 4.535 | 4.718 | 0.001 |
| hsa-miR-5000-5p | 0.010 | 2.048 | 204.820 | 0.000 |
| hsa-miR-500a-5p | 1.202 | 3.657 | 3.044 | 0.013 |
| hsa-miR-518e-5p | 0.010 | 1.024 | 102.410 | 0.031 |
| hsa-miR-519a-5p | 0.010 | 1.756 | 175.560 | 0.002 |
| hsa-miR-519b-5p | 0.010 | 1.024 | 102.410 | 0.031 |
| hsa-miR-519c-5p | 0.010 | 1.024 | 102.410 | 0.031 |
| hsa-miR-522-3p | 0.721 | 2.487 | 3.450 | 0.031 |
| hsa-miR-522-5p | 0.010 | 1.024 | 102.410 | 0.031 |
| hsa-miR-523-5p | 0.010 | 1.024 | 102.410 | 0.031 |
| hsa-miR-542-3p | 29.318 | 36.867 | 1.257 | 0.002 |
| hsa-miR-548aq-3p | 1.682 | 6.291 | 3.740 | 0.000 |
| hsa-miR-548e-5p | 0.010 | 1.317 | 131.670 | 0.008 |
| hsa-miR-548h-5p | 9.613 | 14.191 | 1.476 | 0.009 |
| hsa-miR-548k | 13.458 | 17.263 | 1.283 | 0.031 |
| hsa-miR-548o-3p | 15.861 | 23.261 | 1.467 | 0.001 |
| hsa-miR-548y | 0.010 | 1.170 | 117.040 | 0.016 |
| hsa-miR-550a-5p | 15.140 | 22.676 | 1.498 | 0.001 |
| hsa-miR-561-5p | 356.147 | 496.535 | 1.394 | 0.000 |
| hsa-miR-576-3p | 12.016 | 18.434 | 1.534 | 0.001 |
| hsa-miR-576-5p | 2.884 | 7.608 | 2.638 | 0.000 |
| hsa-miR-579-3p | 0.010 | 2.487 | 248.710 | 0.000 |
| hsa-miR-579-5p | 0.010 | 2.195 | 219.450 | 0.000 |
| hsa-miR-590-3p | 16.822 | 23.261 | 1.383 | 0.002 |
| hsa-miR-616-3p | 0.010 | 1.317 | 131.670 | 0.008 |
| hsa-miR-618 | 0.721 | 4.389 | 6.088 | 0.000 |
| hsa-miR-628-5p | 6.248 | 10.534 | 1.686 | 0.005 |
| hsa-miR-641 | 9.613 | 15.069 | 1.568 | 0.003 |
| hsa-miR-6509-5p | 0.481 | 2.633 | 5.479 | 0.008 |
| hsa-miR-769-5p | 200.903 | 253.973 | 1.264 | 0.000 |
| hsa-miR-7974 | 145.150 | 179.361 | 1.236 | 0.000 |
| hsa-miR-92a-1-5p | 19.225 | 42.134 | 2.192 | 0.000 |
| hsa-miR-93-5p | 606.795 | 621.035 | 1.023 | 0.000 |
| hsa-miR-95-3p | 8.651 | 14.776 | 1.708 | 0.001 |
| hsa-miR-96-5p | 357.829 | 470.202 | 1.314 | 0.000 |
| hsa-miR-99a-5p | 279.726 | 298.009 | 1.065 | 0.000 |
| let-7-x | 958.616 | 1349.013 | 1.407 | 0.000 |
| let-7-y | 9.132 | 13.021 | 1.426 | 0.024 |
| miR-100-x | 838.458 | 1144.196 | 1.365 | 0.000 |
| miR-10174-y | 3.124 | 6.145 | 1.967 | 0.018 |
| miR-101-y | 194.895 | 195.600 | 1.004 | 0.016 |
| miR-103-y | 26.435 | 61.006 | 2.308 | 0.000 |
| miR-10401-x | 0.010 | 1.024 | 102.410 | 0.031 |
| miR-106-x | 15.621 | 27.797 | 1.779 | 0.000 |
| miR-106-y | 3.845 | 6.583 | 1.712 | 0.025 |
| miR-107-y | 22.830 | 58.227 | 2.550 | 0.000 |
| miR-10-x | 91.320 | 255.583 | 2.799 | 0.000 |
| miR-122-x | 0.010 | 1.317 | 131.670 | 0.008 |
| miR-12301-x | 0.481 | 2.341 | 4.871 | 0.013 |
| miR-125-x | 322.502 | 1925.573 | 5.971 | 0.000 |
| miR-125-y | 4.085 | 6.876 | 1.683 | 0.040 |
| miR-1269-y | 6.969 | 10.387 | 1.490 | 0.024 |
| miR-1273-x | 2.644 | 7.900 | 2.989 | 0.000 |
| miR-128-y | 23.311 | 34.380 | 1.475 | 0.000 |
| miR-1296-x | 19.946 | 29.699 | 1.489 | 0.000 |
| miR-1304-x | 1.202 | 3.365 | 2.800 | 0.019 |
| miR-137-y | 0.010 | 1.024 | 102.410 | 0.031 |
| miR-1388-x | 0.010 | 1.170 | 117.040 | 0.016 |
| miR-138-x | 1.923 | 6.730 | 3.500 | 0.000 |
| miR-138-y | 0.010 | 1.024 | 102.410 | 0.031 |
| miR-140-x | 0.010 | 1.170 | 117.040 | 0.016 |
| miR-140-y | 425.838 | 469.616 | 1.103 | 0.000 |
| miR-146-x | 170.624 | 247.390 | 1.450 | 0.000 |
| miR-148-y | 87.715 | 127.279 | 1.451 | 0.000 |
| miR-151-x | 495.529 | 605.088 | 1.221 | 0.000 |
| miR-152-y | 17.543 | 22.823 | 1.301 | 0.011 |
| miR-155-x | 0.010 | 2.926 | 292.600 | 0.000 |
| miR-15-x | 9.372 | 14.484 | 1.545 | 0.004 |
| miR-16-x | 8.411 | 14.776 | 1.757 | 0.001 |
| miR-16-y | 0.721 | 7.900 | 10.959 | 0.000 |
| miR-17-x | 12.496 | 16.678 | 1.335 | 0.022 |
| miR-181-x | 138.662 | 232.760 | 1.679 | 0.000 |
| miR-181-y | 21.869 | 26.480 | 1.211 | 0.020 |
| miR-182-x | 21.628 | 40.671 | 1.880 | 0.000 |
| miR-183-x | 75.699 | 100.653 | 1.330 | 0.000 |
| miR-185-x | 16.101 | 40.525 | 2.517 | 0.000 |
| miR-186-x | 1.442 | 6.145 | 4.261 | 0.000 |
| miR-18-x | 0.010 | 1.170 | 117.040 | 0.016 |
| miR-191-x | 15.140 | 30.430 | 2.010 | 0.000 |
| miR-192-x | 78.102 | 102.555 | 1.313 | 0.000 |
| miR-193-x | 3.845 | 9.509 | 2.473 | 0.000 |
| miR-193-y | 25.473 | 32.917 | 1.292 | 0.002 |
| miR-196-x | 0.010 | 3.804 | 380.370 | 0.000 |
| miR-19-y | 0.481 | 3.804 | 7.914 | 0.000 |
| miR-20-x | 9.132 | 20.043 | 2.195 | 0.000 |
| miR-210-z | 60.559 | 69.930 | 1.155 | 0.001 |
| miR-21-x | 15220.336 | 23883.282 | 1.569 | 0.000 |
| miR-21-y | 197.058 | 207.304 | 1.052 | 0.001 |
| miR-221-x | 57.676 | 81.781 | 1.418 | 0.000 |
| miR-221-y | 33.884 | 74.758 | 2.206 | 0.000 |
| miR-221-z | 2.403 | 6.437 | 2.679 | 0.001 |
| miR-222-y | 2098.669 | 2823.989 | 1.346 | 0.000 |
| miR-224-x | 4.085 | 13.606 | 3.330 | 0.000 |
| miR-22-y | 7.209 | 10.826 | 1.502 | 0.021 |
| miR-2355-x | 4.806 | 9.948 | 2.070 | 0.001 |
| miR-23-y | 1006.438 | 1676.135 | 1.665 | 0.000 |
| miR-24-y | 112.948 | 144.396 | 1.278 | 0.000 |
| miR-26-x | 75.459 | 126.840 | 1.681 | 0.000 |
| miR-26-y | 1.442 | 4.535 | 3.145 | 0.003 |
| miR-27-y | 2621.594 | 3006.861 | 1.147 | 0.000 |
| miR-28-x | 53.350 | 63.054 | 1.182 | 0.001 |
| miR-28-y | 54.552 | 93.484 | 1.714 | 0.000 |
| miR-29-x | 1.202 | 3.950 | 3.287 | 0.005 |
| miR-29-y | 20.907 | 36.721 | 1.756 | 0.000 |
| miR-3001-x | 0.010 | 1.463 | 146.300 | 0.004 |
| miR-30-x | 536.863 | 1005.798 | 1.873 | 0.000 |
| miR-30-y | 72.815 | 106.359 | 1.461 | 0.000 |
| miR-31-x | 222.291 | 1120.350 | 5.040 | 0.000 |
| miR-320-y | 118.235 | 181.995 | 1.539 | 0.000 |
| miR-330-y | 9.613 | 17.263 | 1.796 | 0.000 |
| miR-339-x | 9.132 | 13.313 | 1.458 | 0.015 |
| miR-342-x | 1.682 | 4.389 | 2.609 | 0.009 |
| miR-342-y | 17.543 | 34.965 | 1.993 | 0.000 |
| miR-361-x | 4.326 | 8.485 | 1.962 | 0.003 |
| miR-3622-x | 0.010 | 1.170 | 117.040 | 0.016 |
| miR-365-y | 7.930 | 11.996 | 1.513 | 0.010 |
| miR-378-x | 4.806 | 9.363 | 1.948 | 0.002 |
| miR-378-y | 38.450 | 78.416 | 2.039 | 0.000 |
| miR-3970-x | 0.010 | 1.609 | 160.930 | 0.004 |
| miR-423-x | 11.775 | 19.750 | 1.677 | 0.000 |
| miR-423-y | 26.194 | 37.599 | 1.435 | 0.000 |
| miR-4454-x | 183.360 | 205.549 | 1.121 | 0.000 |
| miR-4484-y | 0.010 | 1.609 | 160.930 | 0.004 |
| miR-452-x | 65.846 | 79.879 | 1.213 | 0.000 |
| miR-452-y | 20.667 | 25.310 | 1.225 | 0.020 |
| miR-484-x | 45.420 | 54.862 | 1.208 | 0.001 |
| miR-484-z | 7.930 | 14.776 | 1.863 | 0.000 |
| miR-491-x | 0.481 | 2.341 | 4.871 | 0.013 |
| miR-497-x | 0.010 | 1.317 | 131.670 | 0.008 |
| miR-500-y | 23.311 | 32.624 | 1.400 | 0.000 |
| miR-502-y | 23.551 | 32.917 | 1.398 | 0.000 |
| miR-532-x | 3.845 | 10.680 | 2.778 | 0.000 |
| miR-548-x | 3.845 | 8.193 | 2.131 | 0.002 |
| miR-548-y | 4.085 | 8.485 | 2.077 | 0.002 |
| miR-561-x | 1.923 | 4.682 | 2.435 | 0.014 |
| miR-589-y | 0.010 | 1.170 | 117.040 | 0.016 |
| miR-6131-y | 1.923 | 6.876 | 3.577 | 0.000 |
| miR-625-x | 0.010 | 1.170 | 117.040 | 0.016 |
| miR-625-y | 19.225 | 34.673 | 1.803 | 0.000 |
| miR-628-y | 5.527 | 8.339 | 1.509 | 0.040 |
| miR-629-x | 3.605 | 7.461 | 2.070 | 0.003 |
| miR-652-y | 3.124 | 6.145 | 1.967 | 0.018 |
| miR-660-x | 0.010 | 4.974 | 497.410 | 0.000 |
| miR-708-x | 5.768 | 10.095 | 1.750 | 0.006 |
| miR-708-y | 0.481 | 13.167 | 27.397 | 0.000 |
| miR-72-x | 70.172 | 90.558 | 1.291 | 0.000 |
| miR-9222-x | 0.010 | 1.170 | 117.040 | 0.016 |
| miR-92-y | 736.325 | 1317.267 | 1.789 | 0.000 |
| miR-93-y | 0.010 | 1.609 | 160.930 | 0.004 |
| miR-941-y | 12.977 | 17.995 | 1.387 | 0.007 |
| miR-96-x | 1.923 | 5.267 | 2.740 | 0.003 |
| miR-99-y | 8.892 | 11.850 | 1.333 | 0.049 |
| novel-m0003-5p | 0.010 | 1.463 | 146.300 | 0.004 |
| novel-m0014-3p | 0.010 | 1.170 | 117.040 | 0.016 |
| novel-m0017-3p | 0.481 | 2.341 | 4.871 | 0.013 |
| novel-m0019-3p | 0.010 | 1.609 | 160.930 | 0.004 |
| novel-m0020-5p | 0.010 | 1.317 | 131.670 | 0.008 |
| novel-m0021-5p | 0.481 | 2.341 | 4.871 | 0.013 |
| novel-m0023-5p | 0.010 | 1.024 | 102.410 | 0.031 |
| novel-m0025-3p | 0.481 | 3.804 | 7.914 | 0.000 |
| novel-m0030-3p | 0.010 | 1.170 | 117.040 | 0.016 |
| novel-m0040-5p | 0.010 | 2.195 | 219.450 | 0.000 |
| novel-m0050-3p | 0.010 | 1.170 | 117.040 | 0.016 |
| novel-m0054-3p | 0.010 | 1.317 | 131.670 | 0.008 |
| novel-m0064-5p | 5.047 | 7.754 | 1.536 | 0.042 |
| novel-m0069-5p | 0.481 | 3.657 | 7.610 | 0.000 |
| novel-m0076-5p | 0.010 | 1.317 | 131.670 | 0.008 |
| novel-m0077-3p | 7.930 | 12.728 | 1.605 | 0.005 |
| novel-m0080-3p | 0.010 | 1.170 | 117.040 | 0.016 |
| novel-m0087-3p | 0.010 | 1.317 | 131.670 | 0.008 |
| novel-m0088-5p | 0.010 | 1.170 | 117.040 | 0.016 |
| novel-m0089-3p | 0.010 | 1.170 | 117.040 | 0.016 |
| novel-m0094-5p | 0.010 | 1.024 | 102.410 | 0.031 |
| novel-m0100-3p | 0.010 | 1.609 | 160.930 | 0.004 |
| novel-m0108-5p | 0.721 | 6.291 | 8.726 | 0.000 |
| novel-m0110-3p | 0.010 | 1.609 | 160.930 | 0.004 |
| novel-m0111-3p | 0.010 | 1.609 | 160.930 | 0.004 |
| novel-m0116-3p | 2.884 | 5.852 | 2.029 | 0.009 |
| novel-m0117-5p | 7.450 | 19.897 | 2.671 | 0.000 |
| novel-m0118-3p | 4.566 | 26.480 | 5.799 | 0.000 |
| novel-m0123-3p | 5.287 | 19.604 | 3.708 | 0.000 |
| novel-m0124-3p | 0.481 | 2.341 | 4.871 | 0.013 |
| novel-m0125-3p | 0.010 | 1.902 | 190.190 | 0.001 |
| novel-m0139-5p | 0.010 | 1.463 | 146.300 | 0.004 |
| novel-m0152-5p | 0.010 | 1.170 | 117.040 | 0.016 |
| novel-m0153-3p | 0.010 | 1.024 | 102.410 | 0.031 |
| novel-m0154-3p | 0.481 | 2.341 | 4.871 | 0.013 |
| novel-m0164-3p | 0.010 | 1.317 | 131.670 | 0.008 |
| novel-m0167-5p | 0.010 | 1.024 | 102.410 | 0.031 |
| novel-m0169-5p | 0.010 | 1.024 | 102.410 | 0.031 |
| novel-m0174-3p | 0.010 | 1.170 | 117.040 | 0.016 |
| novel-m0188-3p | 0.010 | 1.024 | 102.410 | 0.031 |
| novel-m0203-3p | 0.010 | 1.902 | 190.190 | 0.001 |
| novel-m0205-3p | 0.010 | 1.024 | 102.410 | 0.031 |
| novel-m0207-3p | 0.481 | 3.804 | 7.914 | 0.000 |
| novel-m0210-5p | 0.010 | 2.195 | 219.450 | 0.000 |
| novel-m0212-3p | 0.010 | 1.317 | 131.670 | 0.008 |
| novel-m0220-3p | 0.481 | 4.243 | 8.828 | 0.000 |
| novel-m0234-5p | 0.010 | 1.024 | 102.410 | 0.031 |
| novel-m0239-3p | 0.010 | 1.170 | 117.040 | 0.016 |
| novel-m0245-3p | 0.010 | 1.170 | 117.040 | 0.016 |
| novel-m0247-5p | 0.481 | 7.754 | 16.134 | 0.000 |
| novel-m0284-3p | 0.010 | 1.317 | 131.670 | 0.008 |
| novel-m0285-5p | 0.010 | 1.317 | 131.670 | 0.008 |
| novel-m0291-3p | 0.010 | 1.170 | 117.040 | 0.016 |
| novel-m0292-3p | 0.010 | 1.170 | 117.040 | 0.016 |
| novel-m0293-3p | 0.010 | 1.463 | 146.300 | 0.004 |
| novel-m0302-3p | 0.010 | 1.317 | 131.670 | 0.008 |
| novel-m0305-3p | 0.481 | 4.243 | 8.828 | 0.000 |
| novel-m0307-5p | 3.124 | 7.461 | 2.388 | 0.001 |
| novel-m0308-3p | 1.442 | 4.974 | 3.450 | 0.001 |
| novel-m0311-3p | 0.010 | 1.170 | 117.040 | 0.016 |
| novel-m0322-5p | 0.010 | 1.317 | 131.670 | 0.008 |
| novel-m0332-3p | 0.010 | 1.902 | 190.190 | 0.001 |
| novel-m0337-5p | 0.010 | 1.024 | 102.410 | 0.031 |
| novel-m0343-3p | 0.010 | 1.463 | 146.300 | 0.004 |
| novel-m0350-3p | 0.481 | 2.780 | 5.784 | 0.004 |
| novel-m0352-5p | 0.721 | 2.780 | 3.856 | 0.012 |
| novel-m0353-3p | 0.010 | 1.170 | 117.040 | 0.016 |
| novel-m0357-3p | 0.010 | 1.463 | 146.300 | 0.004 |
| novel-m0358-5p | 0.010 | 1.317 | 131.670 | 0.008 |
| novel-m0359-5p | 57.435 | 92.314 | 1.607 | 0.000 |
| novel-m0364-3p | 2.884 | 5.852 | 2.029 | 0.009 |
| novel-m0365-3p | 0.481 | 2.926 | 6.088 | 0.003 |
| novel-m0370-5p | 2.163 | 6.145 | 2.841 | 0.001 |
| novel-m0374-5p | 0.721 | 4.096 | 5.682 | 0.000 |
| novel-m0377-3p | 2.884 | 5.852 | 2.029 | 0.009 |
| novel-m0378-3p | 0.481 | 3.804 | 7.914 | 0.000 |
| novel-m0382-3p | 0.010 | 1.024 | 102.410 | 0.031 |
| novel-m0387-3p | 0.010 | 1.463 | 146.300 | 0.004 |
| novel-m0388-3p | 18.745 | 36.867 | 1.967 | 0.000 |
| novel-m0400-3p | 2.884 | 5.852 | 2.029 | 0.009 |
| novel-m0415-3p | 0.010 | 1.170 | 117.040 | 0.016 |
| novel-m0425-5p | 0.010 | 1.024 | 102.410 | 0.031 |
| novel-m0426-3p | 0.010 | 1.024 | 102.410 | 0.031 |
| novel-m0428-5p | 0.010 | 1.463 | 146.300 | 0.004 |
| novel-m0429-3p | 0.010 | 1.463 | 146.300 | 0.004 |
| novel-m0431-3p | 0.010 | 1.170 | 117.040 | 0.016 |
| novel-m0442-5p | 0.010 | 1.024 | 102.410 | 0.031 |
| novel-m0452-3p | 2.884 | 5.852 | 2.029 | 0.009 |
| novel-m0453-3p | 0.010 | 1.024 | 102.410 | 0.031 |
| novel-m0473-3p | 0.010 | 1.170 | 117.040 | 0.016 |
| novel-m0478-3p | 0.010 | 1.024 | 102.410 | 0.031 |
| novel-m0493-3p | 0.010 | 1.024 | 102.410 | 0.031 |
| novel-m0500-3p | 0.010 | 1.463 | 146.300 | 0.004 |
| novel-m0513-3p | 0.010 | 1.024 | 102.410 | 0.031 |
| novel-m0526-3p | 0.010 | 1.902 | 190.190 | 0.001 |
| novel-m0531-5p | 0.010 | 1.317 | 131.670 | 0.008 |
| novel-m0536-3p | 0.010 | 1.024 | 102.410 | 0.031 |
| novel-m0546-5p | 0.010 | 1.024 | 102.410 | 0.031 |
| novel-m0554-3p | 0.010 | 1.024 | 102.410 | 0.031 |
| novel-m0555-5p | 0.010 | 1.024 | 102.410 | 0.031 |
| novel-m0565-3p | 0.010 | 1.170 | 117.040 | 0.016 |
| novel-m0566-5p | 0.010 | 1.024 | 102.410 | 0.031 |
| novel-m0567-3p | 0.481 | 2.780 | 5.784 | 0.004 |
| novel-m0580-5p | 0.010 | 1.756 | 175.560 | 0.002 |
| novel-m0583-3p | 0.010 | 1.024 | 102.410 | 0.031 |
| novel-m0596-5p | 0.721 | 2.341 | 3.247 | 0.031 |
| novel-m0603-5p | 38.931 | 90.558 | 2.326 | 0.000 |
| novel-m0604-3p | 7.930 | 11.411 | 1.439 | 0.025 |
| novel-m0605-3p | 0.010 | 1.609 | 160.930 | 0.004 |
| novel-m0606-3p | 0.010 | 1.170 | 117.040 | 0.016 |
| novel-m0619-5p | 0.010 | 1.170 | 117.040 | 0.016 |
| novel-m0626-3p | 0.010 | 1.024 | 102.410 | 0.031 |
| novel-m0633-3p | 0.010 | 1.902 | 190.190 | 0.001 |
| novel-m0634-3p | 0.010 | 1.024 | 102.410 | 0.031 |
| novel-m0652-5p | 0.010 | 1.609 | 160.930 | 0.004 |
| novel-m0662-3p | 0.010 | 1.024 | 102.410 | 0.031 |
| novel-m0668-3p | 0.010 | 2.195 | 219.450 | 0.000 |
| novel-m0673-5p | 0.010 | 1.170 | 117.040 | 0.016 |
| novel-m0684-3p | 0.481 | 4.243 | 8.828 | 0.000 |
| novel-m0693-5p | 0.010 | 1.024 | 102.410 | 0.031 |
| novel-m0701-3p | 0.010 | 1.024 | 102.410 | 0.031 |
| novel-m0704-5p | 0.010 | 1.902 | 190.190 | 0.001 |
| novel-m0712-5p | 0.010 | 1.317 | 131.670 | 0.008 |
| novel-m0733-5p | 0.010 | 1.609 | 160.930 | 0.004 |
| novel-m0749-5p | 0.010 | 1.317 | 131.670 | 0.008 |
| Note: NC, negative control; Si, siRNA; TPM, transcripts per million; FC, Fold Change. | | | | |

| Supplementary Table 3. Predicted miRNAs that binds to PCAT6 according to Encyclopedia of RNA Interactomes | | | | | | | | | | |
| --- | --- | --- | --- | --- | --- | --- | --- | --- | --- | --- |
| miRNAname | geneName | geneType | chromosome | start | end | strand | clipExpNum | degraExpNum | RBP | merClass |
| hsa-miR-4731-5p | PCAT6 | antisense | chr1 | 202780086 | 202780108 | + | 1 | 0 | AGO1 | 7mer-m8 |
| hsa-miR-4731-5p | PCAT6 | antisense | chr1 | 202780090 | 202780108 | + | 1 | 0 | AGO1 | 7mer-m8 |
| hsa-miR-1306-5p | PCAT6 | antisense | chr1 | 202780096 | 202780117 | + | 1 | 0 | AGO1 | 7mer-m8 |
| hsa-miR-330-5p | PCAT6 | antisense | chr1 | 202780116 | 202780138 | + | 1 | 0 | AGO1 | 7mer-m8 |
| hsa-miR-326 | PCAT6 | antisense | chr1 | 202780119 | 202780138 | + | 1 | 0 | AGO1 | 7mer-m8 |
| hsa-miR-143-3p | PCAT6 | antisense | chr1 | 202780873 | 202780894 | + | 1 | 0 | AGO1-4 | 7mer-m8 |
| hsa-miR-4770 | PCAT6 | antisense | chr1 | 202780874 | 202780894 | + | 1 | 0 | AGO1-4 | 7mer-m8 |
| hsa-miR-185-5p | PCAT6 | antisense | chr1 | 202780874 | 202780897 | + | 1 | 0 | AGO1-4 | 7mer-m8 |
| hsa-miR-4644 | PCAT6 | antisense | chr1 | 202780874 | 202780897 | + | 1 | 0 | AGO1-4 | 7mer-m8 |
| hsa-miR-6088 | PCAT6 | antisense | chr1 | 202780875 | 202780894 | + | 1 | 0 | AGO1-4 | 7mer-m8 |
| hsa-miR-4784 | PCAT6 | antisense | chr1 | 202780878 | 202780899 | + | 1 | 0 | AGO1-4 | 8mer |
| hsa-miR-4306 | PCAT6 | antisense | chr1 | 202780879 | 202780897 | + | 1 | 0 | AGO1-4 | 7mer-m8 |
| hsa-miR-3150b-3p | PCAT6 | antisense | chr1 | 202780879 | 202780899 | + | 1 | 0 | AGO1-4 | 8mer |
| hsa-miR-543 | PCAT6 | antisense | chr1 | 202780905 | 202780925 | + | 1 | 1 | AGO1-4 | 7mer-m8 |
| hsa-miR-545-3p | PCAT6 | antisense | chr1 | 202780909 | 202780930 | + | 1 | 1 | AGO1-4 | 7mer-m8 |
| hsa-miR-513a-5p | PCAT6 | antisense | chr1 | 202780931 | 202780947 | + | 1 | 0 | AGO1-4 | 8mer |

Supplementary Figure 1


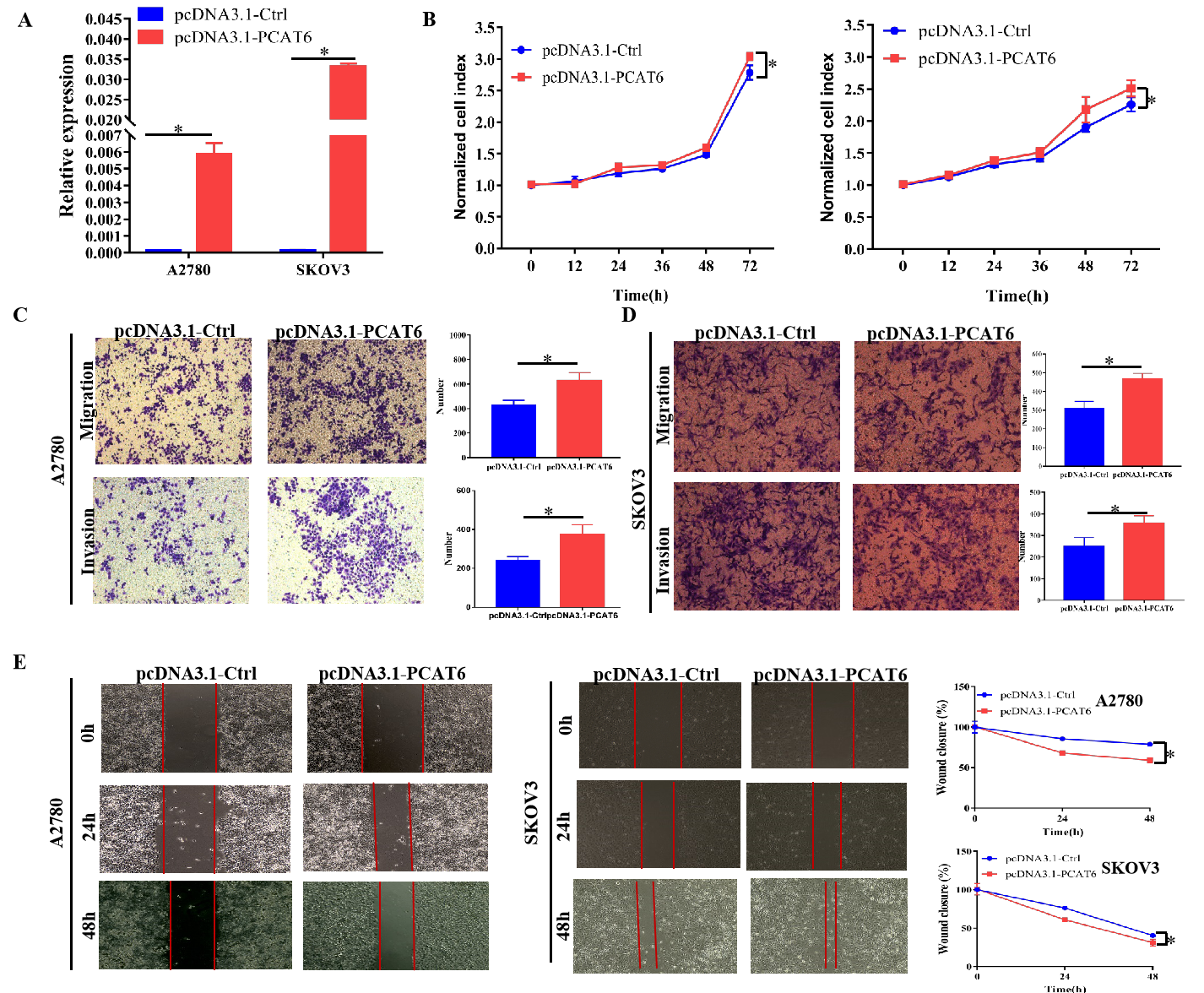


Supplementary Figure 1. Overexpression of PCAT6 in A2780 and SKOV3 cells promoted ovarian cancer cell proliferation, migration and invasion. A. The expression of PCAT6 increased significantly in A2780 and SKOV3 cells after transfection with pcDNA3.1-PCAT6 (pcDNA3.1-PCAT6) compared with the control group (pcDNA3.1-Ctrl). B. CCK-8 assays showed that overexpression of PCAT6 promoted A2780 (left) and SKOV3 (right) cell proliferation. C. Representative images of Transwell migration and invasion assays in A2780 cells (left) after transfection with pcDNA3.1-PCAT6 compared with pcDNA3.1-Ctrl. The mean number of migrated and invaded cells was increased in the pcDNA3.1-PCAT6 group compared with the pcDNA3.1-Ctrl group (right). D. Representative images of Transwell migration and invasion assays in SKOV3 cells (left) after transfection with pcDNA3.1-PCAT6 compared with pcDNA3.1-Ctrl. The mean number of migrated and invaded cells was increased in the pcDNA3.1-PCAT6 group compared with the pcDNA3.1-Ctrl group (right). E. Representative images of wound healing assays in A2780 and SKOV3 cells (left). The motility of ovarian cancer cells was increased in the pcDNA3.1-PCAT6 group compared with the pcDNA3.1-Ctrl group (right). **P* < 0.05.

Supplementary Figure 2


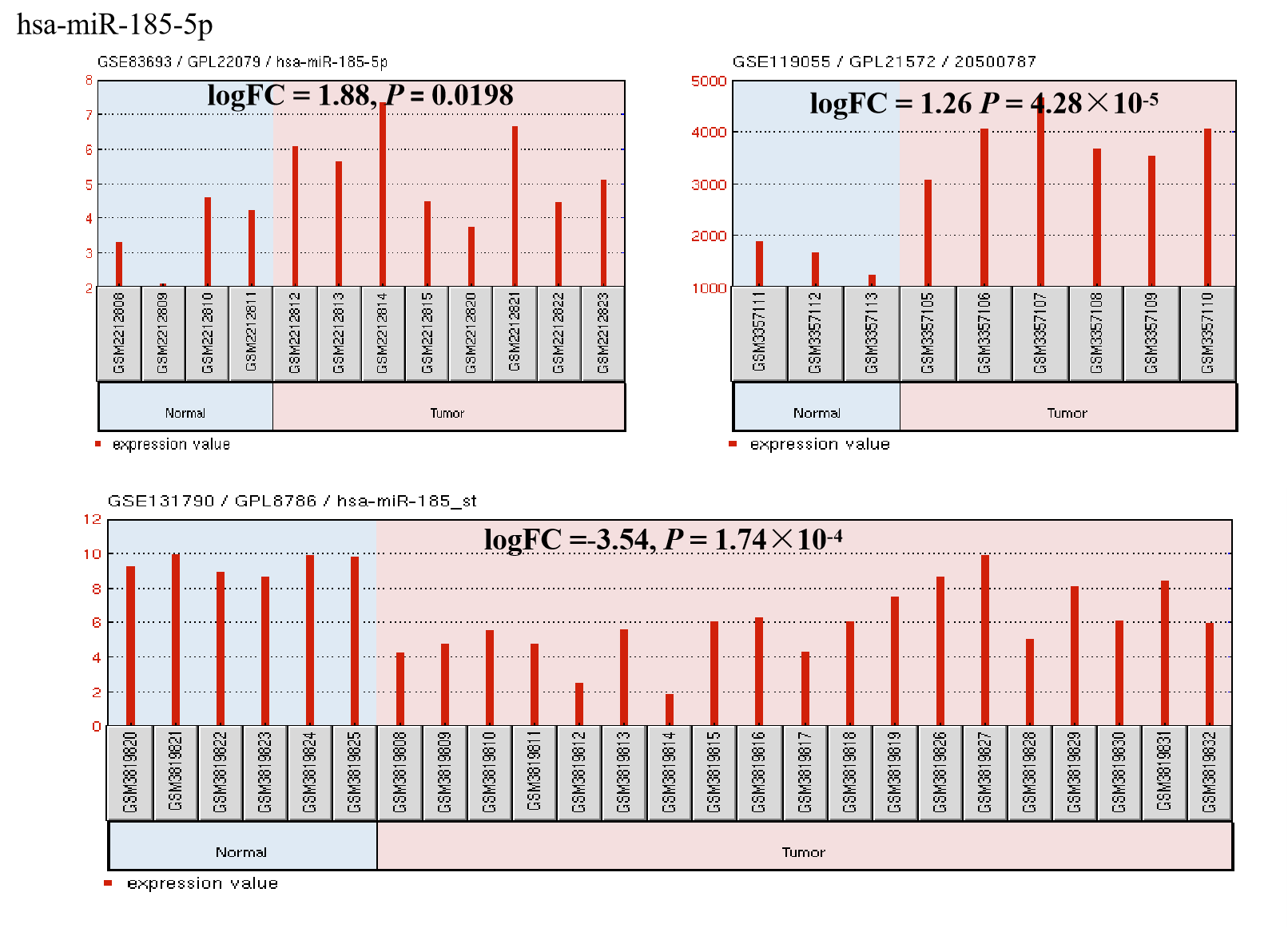


Supplementary Figure 2. miR-185-5p was upregulated in the GSE83693 and GSE119055 datasets but downregulated in the GSE131790 data

Supplementary Figure 3


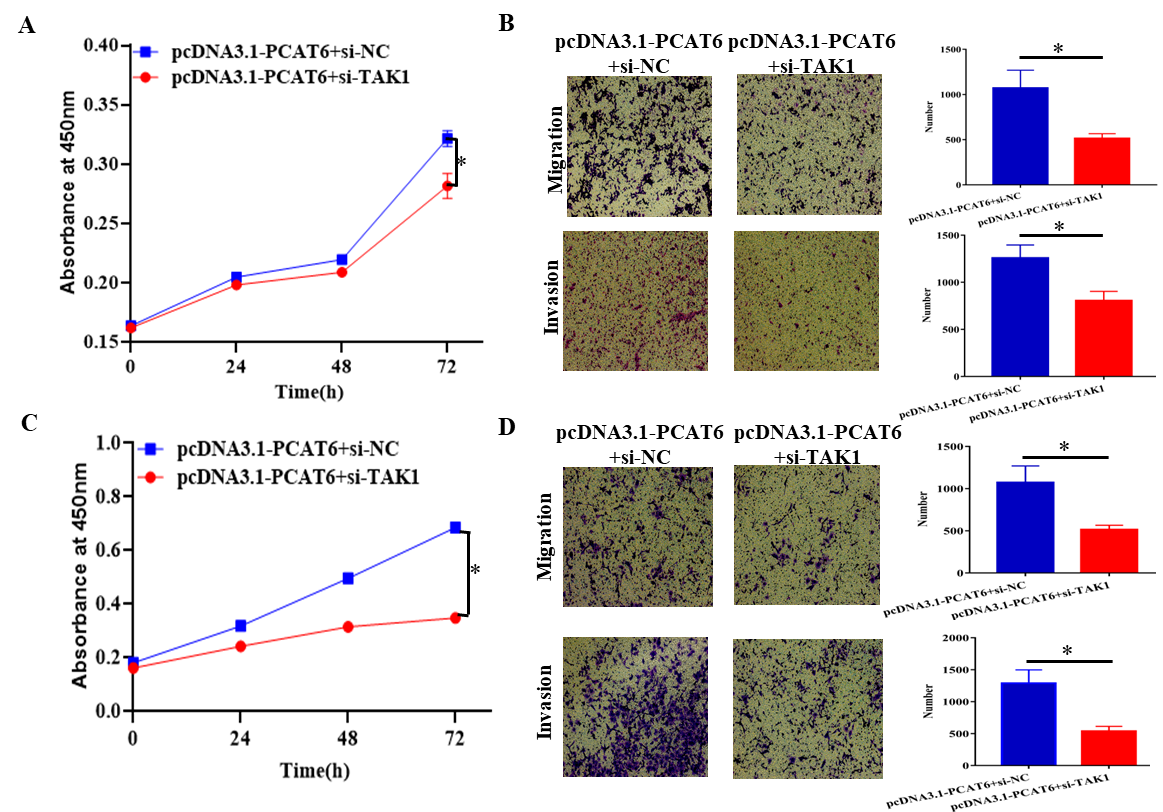


Supplementary Figure 3. Silencing TAK1 inhibited the malignant phenotype of PCAT6 overexpression. A. CCK-8 assays in A2780 cells transfected with pcDNA3.1-PCAT6 and si-TAK1 compared with pcDNA3.1-Ctrl and si-NC. B. Transwell migration and invasion assays in A2780 cells after transfection with pcDNA3.1-PCAT6 and si-TAK1 compared with pcDNA3.1-Ctrl and si-NC. C. CCK-8 assays in SKOV3 cells transfected with pcDNA3.1-PCAT6 and si-TAK1 compared with pcDNA3.1-Ctrl and si-NC. B. Transwell migration and invasion assays in SKOV3 cells after transfection with pcDNA3.1-PCAT6 and si-TAK1 compared with pcDNA3.1-Ctrl and si-NC. **P* < 0.05.
